# Genetic diversity, drug resistance, and transmission patterns of tuberculosis based on whole-genome sequencing in Almaty, Kazakhstan

**DOI:** 10.3389/fmicb.2025.1649137

**Published:** 2025-09-16

**Authors:** Nurlan Takenov, Alibi Kaziyev, Asfendiyar Mukhamadi, Lyailya Chingissova, Bekzat Toxanbayeva, Venera Bismilda, Malik Adenov, Lyazzat Eralieva, Narymzhan Nakisbekov, Gulnur Zhunussova

**Affiliations:** ^1^National Tuberculosis Reference Laboratory, National Scientific Center of Phthisiopulmonology of the Republic of Kazakhstan, Almaty, Kazakhstan; ^2^Faculty of Biology and Biotechnology, Al-Farabi Kazakh National University, Almaty, Kazakhstan; ^3^Institute of Genetics and Physiology CS MSHE RK, Almaty, Kazakhstan

**Keywords:** tuberculosis, whole-genome sequencing (WGS), drug resistance, transmission, *Mycobacterium tuberculosis*, phylogenetic diversity, Kazakhstan

## Abstract

Tuberculosis, particularly multidrug-resistant TB (MDR-TB), remains a major public health concern in Kazakhstan, where 26% of new TB cases are MDR, far exceeding the global average. To better understand the genetic diversity, drug resistance, and transmission dynamics of *Mycobacterium tuberculosis* in Kazakhstan, we conducted a retrospective study at the National Scientific Center of Phthisiopulmonology in Almaty from 2023 to 2024. Whole-genome sequencing (WGS) was performed on 272 culture-confirmed TB isolates collected from patients across the country. Phylogenetic analysis revealed the predominance of Lineage 2 (East Asian genotype, 72.4%) and Lineage 4 (Euro-American genotype, 26.8%). Drug resistance profiling identified 29.0% of isolates as MDR-TB, of which 3.3% were classified as pre-XDR and 0.7% as XDR. The most frequently observed resistance-associated mutations were *katG* S315T (99.2%) and *rpoB* S450L (91.1%). Cluster analysis using a ≤ 12 SNP threshold identified 22 genomic clusters involving 80 isolates (29.4%), indicating recent and possibly ongoing transmission. Spatial mapping showed that nearly 60% of clusters spanned multiple regions, while others were highly localized, suggesting household or close-contact transmission. A Mantel correlogram test revealed a statistically significant correlation between geographic and genomic SNP distances in Almaty and Almaty Region (r = 0.0634, *p* = 0.041) within the first distance class (average 5 km, range 0–8 km). These findings suggest that patients living in close proximity are more likely to carry genetically similar strains. As distance increases, geographic proximity becomes less predictive of transmission, with other factors—such as mobility, shared environments, or healthcare contact—likely playing a greater role. Our findings underscore the need to integrate WGS into national TB control programs to guide targeted interventions, enhance surveillance, and curb the spread of drug-resistant TB strains across Kazakhstan.

## Introduction

Tuberculosis (TB) is a preventable and curable infectious disease that affects millions of people every year. TB is caused by bacillus *Mycobacterium tuberculosis,* which is spread through air. The disease typically affects the lungs (pulmonary TB) but can also manifest in other sites within the body. According to the World Health Organization (WHO) reports in 2024, an estimated 10.8 million people developed TB (incident cases) with 400,000 people developing multidrug-resistant TB (MDR-TB) in 2023 (World Health Organization, 2024).

Despite the gradual decline in TB incidence rates year after year, Kazakhstan is facing an epidemic of MDR-TB (World Health Organization, n.d.). In contrast to the global average where MDR-TB represents 3.2% among new TB cases, in Kazakhstan, 26% of newly diagnosed TB cases are attributed to MDR-TB (World Health Organization, 2024; Authors, n.d.-a). Moreover, Kazakhstan remains on the WHO’s list of the top 30 high MDR-TB burden countries worldwide (World Health Organization, 2023a; World Health Organization, 2024). Hence understanding the factors driving MDR-TB prevalence in Kazakhstan and developing targeted strategies to mitigate this epidemic is crucial.

Previous studies have shown that the transmission of MDR-TB strains is a driver of MDR-TB epidemics in the former Soviet Union countries including Kazakhstan (Auganova et al., 2023; Merker et al., 2018). Therefore, it is of great significance to understand the transmission and risk factors of MDR-TB acquisition in the country for the formulation of the anti-TB policies. Whole-genome sequencing (WGS) is a novel technique that could provide new insights of TB and address the problem. WGS of *M. tuberculosis* is widely used to predict its drug resistance, perform phylogenetic classification, investigate transmission chains, identify mixed infections, and reveal the evolution of the pathogen (Satta et al., 2018; Liu et al., 2022). Only a few studies have used WGS to investigate and characterize TB and MDR-TB strains in the country, primarily focusing on the largest cities, Astana and Almaty (Auganova et al., 2023; Tarlykov et al., 2020; Daniyarov et al., 2021; Auganova et al., 2023; Daniyarov et al., 2020; Daniyarov et al., 2023; Kairov et al., 2014). However, comprehensive nationwide studies utilizing WGS to assess the genetic diversity, transmission dynamics, and drug resistance of *M. tuberculosis* strains across different regions of Kazakhstan remain limited (Auganova et al., 2023). To provide a scientific basis for DR-TB control and prevention, we conducted a retrospective cohort study on TB from 2023 to 2024, using the WGS to better understand the drug-resistance, genetic diversity, transmission dynamics.

## Methods

### Study setting

The study was conducted at the National Scientific Center of Phthisiopulmonology (NSCP) under the Ministry of Health of the Republic of Kazakhstan, located in Almaty. The NSCP is a leading institution for TB prevention, diagnosis, and treatment, admitting patients from across the country. This retrospective study was based on data collected from January 2023 to December 2024 at the National Tuberculosis Reference Laboratory.

For bacteriological confirmation, biological specimens from symptomatic patients were submitted to the laboratory before initiation of treatment. TB diagnosis followed the official protocol outlined in Order of the Minister of Healthcare of the Republic of Kazakhstan dated November 30, 2020, № RK DSM-214/2020 “On Approval of the Rules for Conducting Tuberculosis Prevention Activities” (Authors, n.d.-b). Patients were eligible for the study if they tested positive for both the Xpert MTB/RIF assay and culture using the Mycobacteria Growth Indicator Tube (MGIT) system. Informed consent was obtained from patients or legal guardians after providing detailed information about the study objectives. Patient data were extracted from the Damumed Integrated Medical Information System and the National Tuberculosis Patient Registry. These included demographic variables (sex, age, place of residence, and nationality) and clinical characteristics (history of TB treatment, concomitant disease, BMI, and HIV status).

### Sample processing and culture procedures

During routine diagnostic work-up, each sputum specimen was subjected to both molecular and culture-based testing. The Xpert MTB/RIF assay (Cepheid, Sunnyvale, CA, USA) was performed according to the manufacturer’s instructions for rapid detection of *M. tuberculosis* complex and rifampicin resistance. In parallel, sputum samples were liquefied and decontaminated using the N-acetyl-L-cysteine–sodium hydroxide method (Ratnam et al., 1987). Following decontamination, 0.5 ml of the processed sputum was inoculated into MGIT culture tubes and incubated at 37°C in the BACTEC MGIT 960 system (Becton Dickinson, Franklin Lakes, NJ, USA) to promote the growth and detection of *M. tuberculosis*. From the resulting positive MGIT cultures, a 0.5 ml aliquot of broth with sediment containing clinical strains was transferred into a cryovial using a pipette and stored at −80°C until further use for WGS. To obtain pure *M. tuberculosis* biomass for DNA extraction, sediment from MGIT cultures was re-cultured on Löwenstein–Jensen (LJ) solid medium and incubated at 37°C for up to 4 weeks.

### WGS and data analysis

Genomic DNA was extracted and purified using the PureLink Genomic DNA Mini Kit (Invitrogen, Waltham, USA). DNA concentration was measured using both a NanoDrop spectrophotometer and the Qubit dsDNA HS Assay Kit (Thermo Fisher Scientific, Waltham, USA). A DNA concentration range of 0.2–0.4 ng/μL was used for sequencing library preparation with the Illumina Nextera XT Library Preparation Kit (Illumina, San Diego, USA). Whole-genome sequencing was performed on the Illumina MiSeq platform, generating paired-end FASTQ files.

Raw sequencing reads were processed using TBProfiler (v6.6.3), a tool that identifies *M. tuberculosis* lineages, drug resistance-associated mutations, and other genomic features (Phelan et al., 2019). Lineage assignment was carried out within TBProfiler using a validated SNP-based barcoding scheme, with alignment to the *M. tuberculosis* H37Rv reference genome (GenBank accession number NC_000962.3) under default parameters. For additional validation and phylogenetic tree construction, MTBseq (v.1.0.3) was employed (Kohl et al., 2018). A phylogenetic tree was constructed using the maximum likelihood method implemented in IQ-TREE2 (Minh et al., 2020) and visualized using iTOL^1^.

The drug resistance spectrum of each strain was predicted based on 16 anti-tuberculosis drugs using the latest WHO-recommended mutation catalogue (World Health Organization, 2023b). Only group 1 (associated with resistance) and group 2 (associated with resistance—interim) mutations were used for resistance prediction. Drug resistance classes were defined according to the updated 2021 WHO definitions. HR-TB was defined as resistance to isoniazid. MDR-TB was defined as resistance to at least isoniazid and rifampicin. Pre-extensively drug-resistant tuberculosis (pre-XDR-TB) was defined as MDR-TB with additional resistance to any fluoroquinolone. Extensively drug-resistant tuberculosis (XDR-TB) was defined as MDR-TB with additional resistance to any fluoroquinolone and at least one additional Group A drug (bedaquiline or linezolid; World Health Organization, 2021). The genetic distance was calculated to analyze TB transmission characteristics in the study areas, and genomic transmission clusters were defined using 12 SNPs as cutoff values.

### Statistical analysis

SPSS version 27 software (SPSS Inc., Chicago, Illinois) and R version 4.3.1 (RStudio, PBC, Boston, MA, USA) were used for the statistical analysis. Mantel and Mantel correlogram tests were used to assess correlations between continuous variables (geographic distances and SNP differences) across sample pairs. The test employed Pearson’s correlation coefficient to assess the association between genetic and spatial distances. Results with a *p*-value less than 0.05 were considered statistically significant.

## Results

### Demographic and clinical characteristics

**Figure 1:**
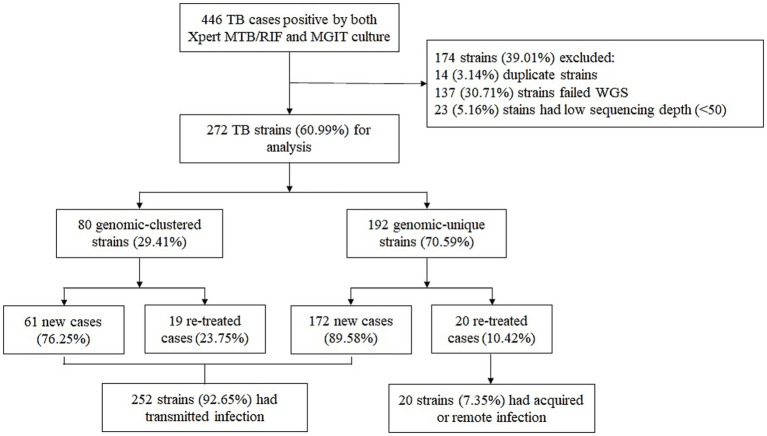
Classification of TB cases based on treatment history and genomic analysis. WGS, whole-genome sequencing.

A total of 446 TB-positive cases, confirmed by Xpert MTB/RIF and MGIT, were collected between January 2023 and December 2024 at the NSCP in Almaty, Kazakhstan. After excluding 14 duplicate strains collected from the same patients, 432 representative *M. tuberculosis* strains were selected for WGS. Of these, WGS was performed on 295 strains, while the remaining 137 were not sequenced due to the lack of culturable *M. tuberculosis*, probably caused by sample degradation, poor bacterial growth and contamination. Additionally, 23 strains had low genome coverage (<50×) and were excluded from the study. Overall, 272 (63.0%) *M. tuberculosis* clinical isolates were successfully sequenced and included in the final analysis (Figure 1). Most patients diagnosed with TB (57.35%, 156/272) were male. The adolescent accounted for 10.29% (28/272) of the study population. The mean age was 40.56 years (range, 1–87 years), and the percentage of patients of Kazakh nationality was 72.79% (198/272). Moreover, most patients were residents of Almaty-city and Almaty region (52.94%, 144/272), and 141 patients (51.84%, 141/272) had no any comorbidities. Pulmonary TB was diagnosed in 229 (84.19%) patients, and extrapulmonary TB was found in the other 43 (15.81%). Most patients (85.66%, 233/272) were newly diagnosed, whereas 39 (14.34%) had previously received anti-TB treatment. The percentage of patients with HIV-positive status was 8.82% (24/272). The average BMI index was 21 (range, 11.8–42.2). The detailed demographic information and clinical characteristics of the study population are presented in Table 1.

**Table 1:** Demographic information and clinical characteristics of 272 patients infected with TB.

Characteristics	Total (*n* = 272) *n* (%)	New cases (*n* = 233) *n* (%)	Retreated (*n* = 39) *n* (%)
Sex			
Male	156 (57.4)	134 (57.5)	22 (56.4)
Female	116 (42.6)	99 (42.5)	17 (43.6)
Age group			
<18	28 (10.3)	26 (11.2)	2 (5.1)
18–29	58 (21.3)	52 (22.3)	6 (15.4)
30–39	45 (16.5)	38 (16.3)	7 (17.9)
40–49	59 (21.7)	50 (21.5)	9 (23.1)
50–59	36 (13.2)	27 (11.6)	9 (23.1)
>60	46 (16.9)	41 (17.6)	5 (12.8)
Nationality			
Kazakh	198 (72.8)	168 (72.1)	30 (76.9)
Other	74 (27.2)	65 (27.9)	9 (23.1)
Clinical form			
Pulmonary	229 (84.2)	193 (82.8)	36 (92.3)
Extrapulmonary	43 (15.8)	40 (17.2)	3 (7.7)
Comorbidities			
HIV	24 (8.8)	21 (9.0)	3 (7.7)
Diabetes	26 (9.6)	21 (9.0)	5 (12.8)
Other/None	222 (81.6)	191 (82.0)	31 (79.5)

### Lineage distribution

**Figure 2:**
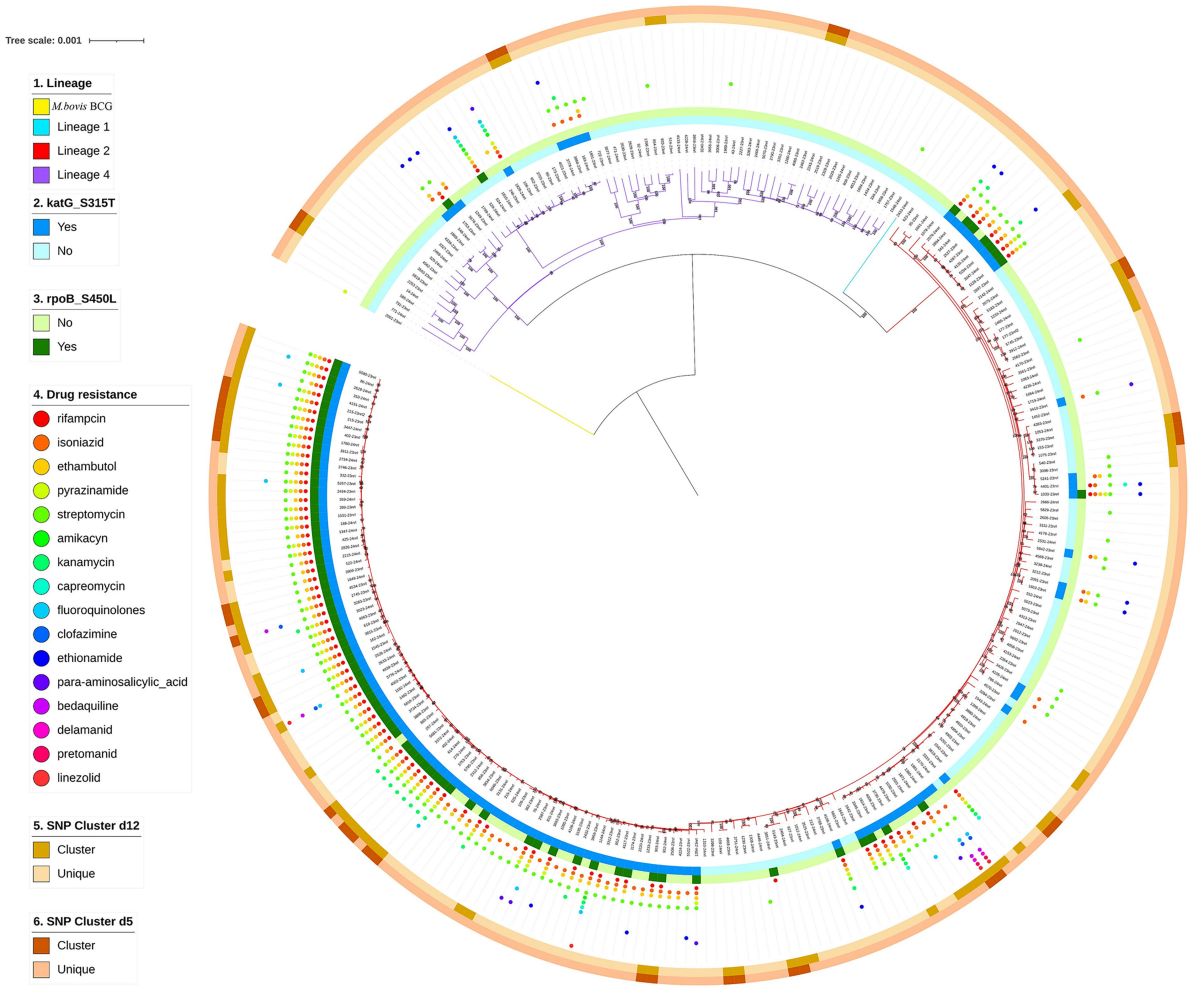
Phylogenetic tree of 272 TB strains isolated in NSCP, Almaty. Note the different colors on the branches indicate different lineages. The first outer circle indicates the presence or absence of *katG* S315T. The second outer circle indicates the presence or absence of *rpoB* S450L. The small circles of different colors on the outer middle ring indicate drug resistance. The third outer circle indicates genomic-clustered strains differing by ≤12 SNPs. The outer-most circle indicates genomic-clustered strains differing by ≤5 SNPs.

A phylogenetic tree was constructed based on whole-genome sequences of 272 *M. tuberculosis* clinical isolates from NSCP in Almaty. Two major lineages were identified: specifically, 72.43% (197/272) *M. tuberculosis* isolates were assigned to lineage 2 (East Asian genotype), and 26.84% (73/272) were assigned to lineage 4 (Euro-American genotype). Additionally, one isolate (0.37%; 1/272) isolates were determined as lineage 1 (Indo-Oceanic genotype), and one was identified as *Mycobacterium bovis* BCG, recovered from a 1-year-old infant with immunodeficiency who developed TB following BCG vaccination. Regarding sublineages, the majority (72.43%, 197/272) belonged to lineage 2.2.1. The remaining 27 isolates belonged to lineage 4.8 (9.93%, 27/272); 16 isolates belonged to lineage 4.3.3 (5.88%, 16/272), and eight isolates belonged to lineage 4.5 (2.94%, 8/272). A maximum likelihood phylogeny of the 272 *M. tuberculosis* isolates, calculated using a concatenated list of 13,844 SNPs (Figure 2), fully confirmed the phylogenetic classification of the strains investigated.

### Resistance profile

**Figure 3:**
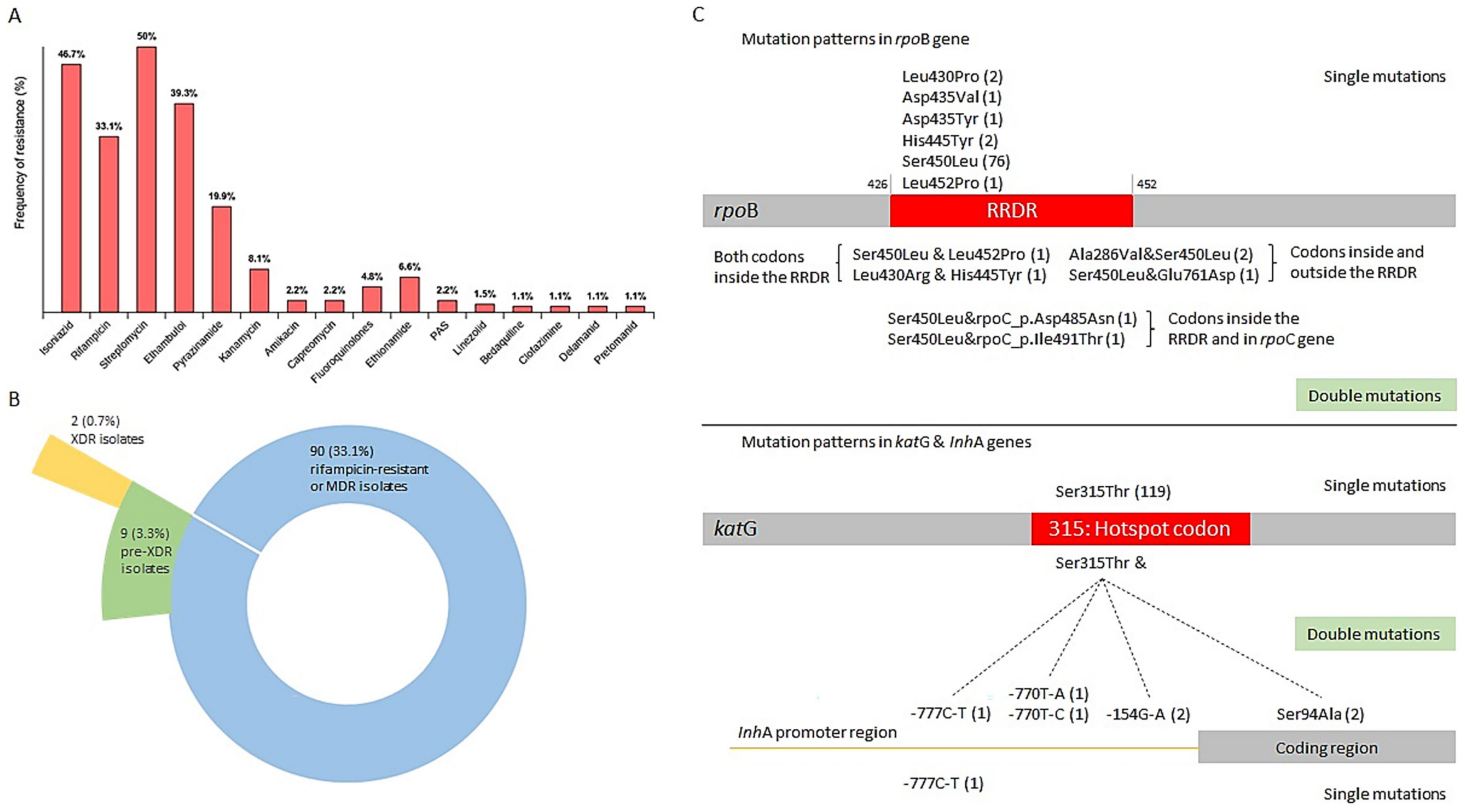
Classification of drug resistance and mutation patterns in the *rpoB* and *katG* genes. **(A)** The frequency of resistance to each of the 16 drugs. **(B)** Pie chart showing the distribution of MDR or rifampicin-resistant, pre-XDR and XDR isolates. **(C)** Mutation patterns in the *rpoB* and *katG* genes. The number of strains is indicated in parentheses. MDR, multidrug resistant. Pre-XDR, pre-extensively drug resistant.

Resistance to 16 anti-TB drugs was detected, with the overall rates (including both new and recurrent TB cases) ranked from highest to lowest as follows: streptomycin (SM, 136/272, 50%), isoniazid (INH, 127/272, 46.69%), ethambutol (EMB, 107/272, 39.34%), rifampicin (RIF, 90/272, 33.09%), pyrazinamide (PZA, 54/272, 19.85%), kanamycin (KM, 22/272, 8.09%), ethionamide (ETO, 18/272, 6.62%), fluoroquinolones (FQs, 13/272, 4.78%), amikacin (AM, 6/272, 2.21%), capreomycin (CM, 6/272, 2.21%), para-aminosalicylic acid (PAS, 6/272, 2.21%), linezolid (LZD, 4/272, 1.47%), bedaquiline (BDQ, 3/272, 1.1%), clofazimine (CFZ, 3/272, 1.1%), delamanid (DEL, 3/272, 1.1%), pretomanid (PTO, 3/272, 1.1%). Strains resistant to cycloserine (Cs) were not detected. The rates of HR-TB, MDR-TB, pre-XDR, XDR strains were 13.97% (38/272), 29.04% (79/272), 3.31% (9/272), and 0.74% (2/272), respectively. Remaining 130 *M. tuberculosis* isolates (130/272, 47.8%) showed no resistance mediating mutations and were classified as drug susceptible. The distribution of resistance classes across major *M. tuberculosis* lineages is summarized in Table 2. A follow-up genetic analysis of drug resistance-associated mutations revealed that the most common mutation in RIF-resistant strains was *rpoB* S450L (82/90, 91.11%), while INH-resistant isolates most frequently carried the *katG* S315T mutation (126/127, 99.21%; Figure 3). In BDQ- and CFZ-resistant strains, two harbored the mutation *mmpR5* c.139dupG, and one carried *mmpR5* c.466dupC. Among the three DEL-resistant strains, each exhibited a unique mutation: *ddn* c.223delG, *ddn* p. Glu118*, and *fbiC* c.2565_*56delCNNNNNNNNNNNNNNNNNNNNNNNNNNNNNNNNNNNNNNNNNNNNNNNNNNNNNNNNNN.

**Table 2:** Drug resistance and lineage classification of *M. tuberculosis* strains in Kazakhstan.

Lineage	No. of Isolates	Drug-susceptible	HR-TB	MDR-TB	Pre-XDR-TB	XDR-TB	Non-MDR resistance^1^
		*n* (%)	*n* (%)	*n* (%)	*n* (%)	*n* (%)	*n* (%)
Lineage 2	197	69 (35)	32 (16.2)	78 (39.6)	7 (3.6)	2 (1)	9 (4.6)
Lineage 4	73	59 (80.8)	6 (8.2)	1 (1.4)	2 (2.7)	0	5 (6.8)
Total	270	128 (47.4)	38 (14.1)	79 (29.3)	9 (3.3)	2 (0.7)	14 (5.2)

^1^Includes isolates resistant to one or more anti-TB drugs (e.g., streptomycin, ethambutol) that do not meet the WHO definitions for MDR, pre-XDR, or XDR-TB. *Two isolates—one *M. bovis* BCG strain and one lineage 1 *M. tuberculosis* isolate—were excluded from this analysis. Both were drug-susceptible and not included in lineage-specific comparisons.

### Clustering analysis

Overall, 80 *M. tuberculosis* strains were divided into 22 clusters, ranging in size from two to 18 strains, suggesting recent transmission. The cluster rate, defined as the proportion of isolates within SNP-defined transmission clusters (≤12 SNPs), was 29.41% (80/272). The median SNP distance of clustered strains were 8.5, and 90% (72/80) of them were Lineage 2, and all drug resistant strains were also L2 strains (Table 3). Moreover, most clusters contained two strains, accounting for 32.5% of the clustered cases (26/80). A previous study showed that the presence of TB among new cases suggests the transmission of TB strains (Yang et al., 2017). Therefore, cases of newly diagnosed TB were combined with those in the genomic clusters, indicating that 87.87% (239/272) of the cases were likely caused by transmission in our study (Figure 1).

**Table 3:** Drug-resistance of clustered *M. tuberculosis* strains stratified by lineage.

Lineage	No. of clusters	Clustered isolates	Median SNP distance	Drug resistance					
				Drug susceptible	HR-TB	MDR	Pre-XDR	XDR	Non-MDR
L 2	18	72	10	18	5	41	5	1	2
L4	4	8	2	8	0	0	0	0	0
Total	22	80	8.5	26	5	41	5	1	2

The results of the maximum-likelihood tree of clustered strains showed 22 clusters including the largest cluster (C18) of strains spanning the study period (2023–2024). In total, 22.73% of the clusters (5/22) included strains with different drug-resistance spectra. Drug-resistance profiles progressively increased in the strains of two clusters (C7, C20) in relation to chronology, including the C7 cluster, with increased drug-resistance profiles against RIF, the C20 cluster, against PZA, and FQs. The ancestral strains from clusters C6 and C8 showed broader drug-resistance profiles than their descendants. In the largest cluster C18, all strains exhibited an identical drug-resistance profile against RIF, INH, PZA, EMB and STR, with only two harboring additional mutations conferring resistance to FQs.

### Spatial distribution of clustered patient samples

**Figure 4:**
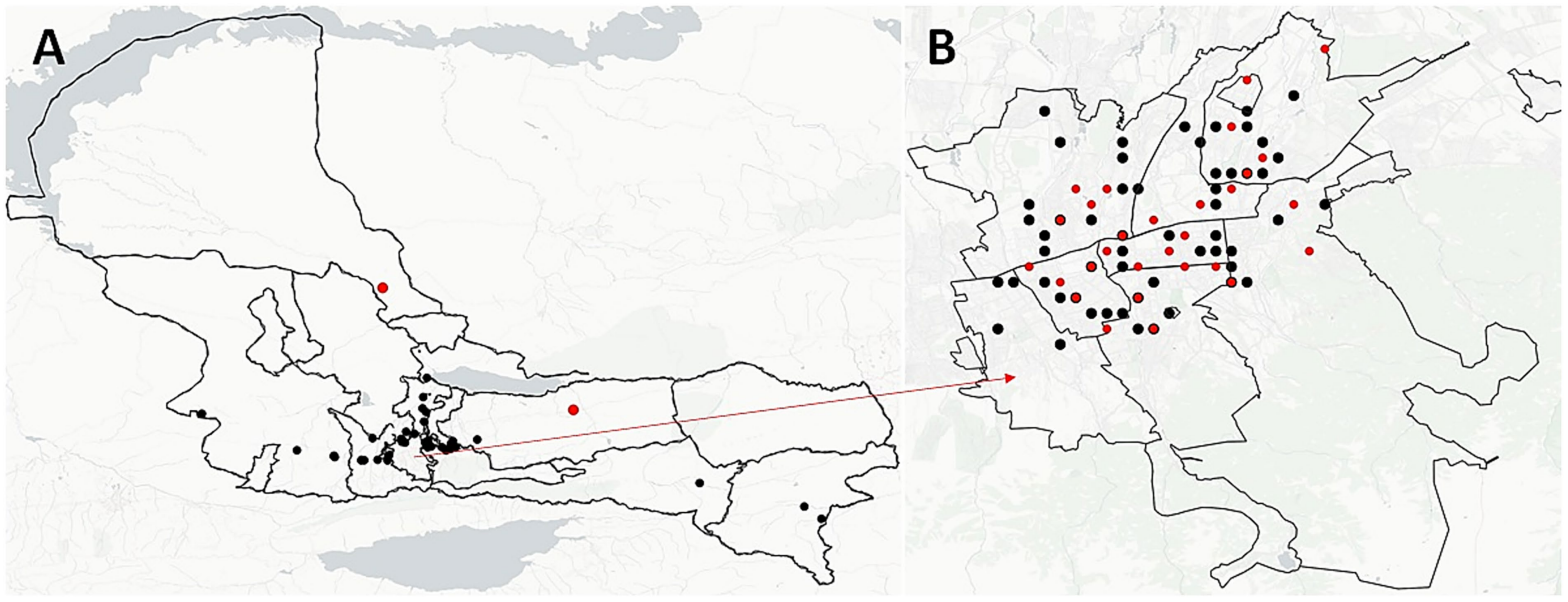
Spatial distribution of unique and clustered *M. tuberculosis* strains in Almaty region **(A)** and Almaty-city **(B)**. Black dots illustrate the spatial location of each strain and red dots illustrate the location of clustered strains.

We analyzed the geographic distribution of patient residential addresses across all 22 identified clusters. Four clusters (18.2%) consisted of patients residing in the same town, five clusters (22.7%) included patients from different towns within the same region, and 13 clusters (59.1%) spanned different regions entirely. In the largest cluster, C18, half of the patients (9 out of 18) were from Almaty, with five of them residing within a 5 km radius of each other (Figure 4, red dots). Notably, in cluster C3, all patients lived in the same residential building, differing only by apartment number—strongly suggesting potential for close-contact transmission.

**Figure 5:**
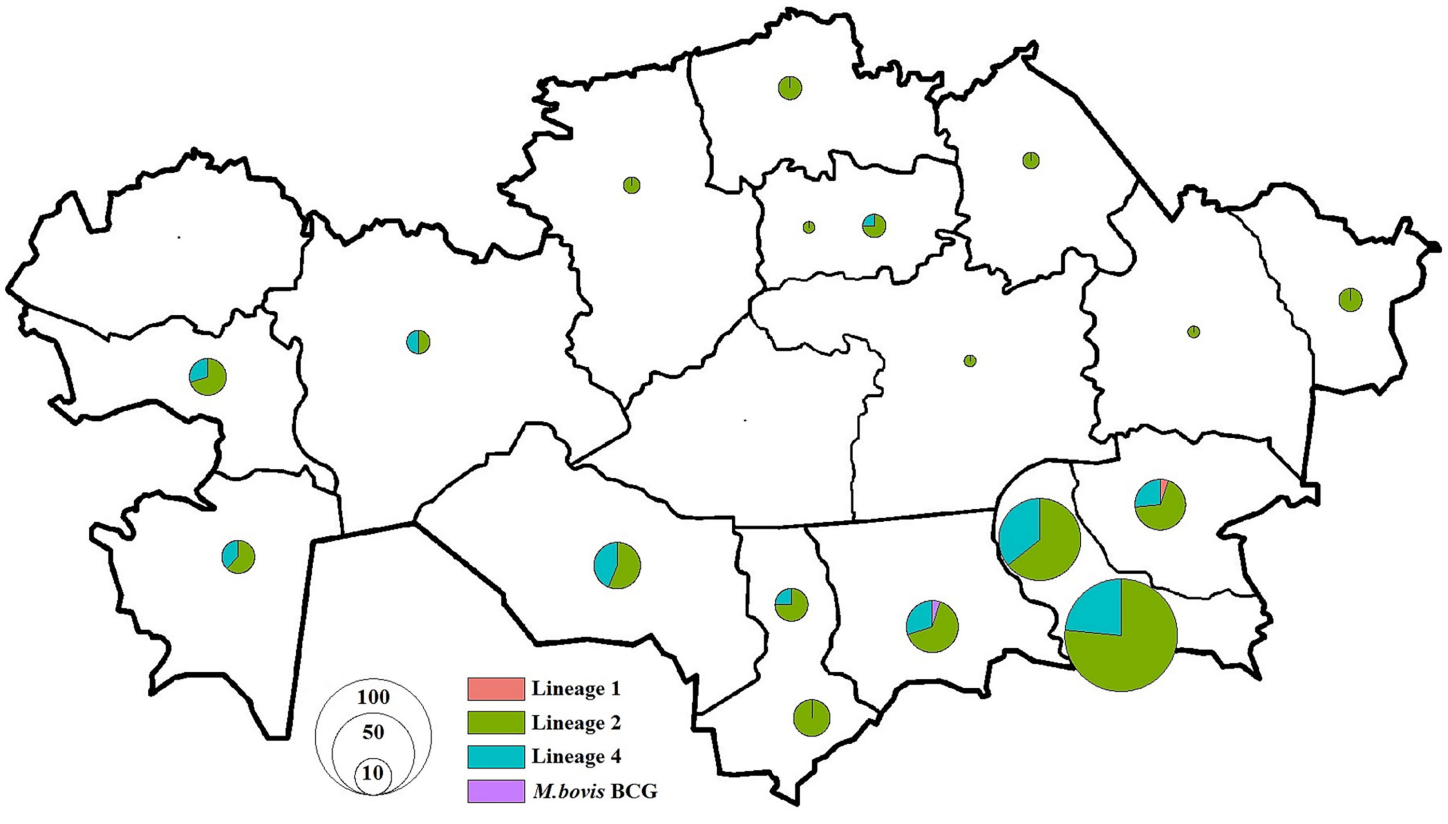
Distribution of *M. tuberculosis* isolates in Kazakhstan used in this study. Pie charts show the proportion of isolates among different lineages for each location. The size of pie charts corresponds with the number of isolates.

To investigate whether geographic proximity was associated with genetic similarity of *M. tuberculosis* strains, we performed a geospatial correlation analysis using the Mantel test based on pairwise SNP distances. This analysis was restricted to residential addresses in Almaty and Almaty Region, which accounted for over 50% of the total dataset (Figures 4, 5). Limiting the analysis to this subset ensured sufficient data density and minimized confounding due to regional variability in transmission dynamics and healthcare infrastructure.

The global Mantel test revealed no statistically significant correlation between geographic distance and genetic distance among *M. tuberculosis* strains (Mantel statistic r = 0.06173, *p* = 0.1628), suggesting that, overall, closer residential proximity did not correspond to greater genetic similarity.

However, a more detailed spatial autocorrelation analysis using the Mantel correlogram uncovered significant correlations at specific distance classes. Significant positive correlation was observed in the first distance class (D.cl.1; Mantel r = 0.0634, *p* = 0.041), indicating that patients living very close to each other (within ~5 km; range 0–8 km) were more likely to carry genetically similar strains.

## Discussion

To the best of our knowledge, this is the first comprehensive molecular epidemiological study using WGS to characterize *M. tuberculosis* genetic diversity, drug resistance, and transmission dynamics over a two-year period in Kazakhstan. Among the 272 successfully sequenced *M. tuberculosis* isolates, the dominant lineage was lineage 2.2.1 (East Asian genotype), accounting for 72.43% of cases, while the clustering rate—indicating potential recent transmission—was 29.41%. Notably, 92.65% (252/272) of TB cases were associated with recent transmission, as inferred by genomic clustering and case status, highlighting ongoing community-level spread.

Overall, 52.2% (142/272) of isolates harbored resistance to at least one anti-TB drug, and 29.04% (79/272) met the definition of MDR-TB. Importantly, 13.97% (38/272) of cases were HR-TB, underscoring the clinical significance of detecting isoniazid resistance early—especially as this resistance is not identified by the widely used GeneXpert MTB/RIF assay. In such cases, treatment regimens may be suboptimal, facilitating ongoing transmission and further resistance acquisition. These findings support the urgent need for the broader implementation of diagnostics like the cobas assay (Roche), the BD MAX (Becton Dickinson) and the Xpert MTB/XDR (Cepheid), which detects specific canonical isoniazid resistance conferring mutations and thus improve HR-TB case detection (Klopper et al., 2024).

Consistent with global patterns, the most frequent mutations observed were *katG* S315T (99.21%, 126/127) for INH resistance and *rpoB* S450L (91.11%, 82/90) for RIF resistance. The *rpoB* S450L mutation is causing the least fitness loss, often accompanied by compensatory mutations in *rpoA* or *rpoC*, which can fully restore fitness (Lempens et al., 2023). The strikingly high prevalence of *katG* S315T mutation in Kazakhstan compared to other regions (Australia: 65.4%, India: 71%, China: 63%, Iran: 53.3%) suggests a dominant, actively transmitted INH-resistant lineage (Sailo et al., 2022). While data from neighboring Central Asian countries is sparse, our findings likely reflect regional trends and emphasize the necessity of expanded molecular surveillance across the region (Auganova et al., 2023; Daniyarov et al., 2021).

Globally, the majority of studies frequently have shown that mutations within the 81-bp rifampicin-resistance-determining region (RRDR) of the *rpoB* gene account for over 95% of RIF resistance (Sailo et al., 2022; Nono et al., 2025). In this study, all RIF-resistant strains carried mutations within the 81-bp RRDR of the *rpoB* gene, with the S450L substitution dominating.

Importantly, pre-XDR-TB and XDR-TB were identified in 3.31 and 0.74% of isolates, respectively—rates notably higher than the global averages reported in the 2024 WHO TB report. Mutations associated with resistance to bedaquiline and clofazimine were predominantly found in *mmpR5*, including c.139dupG and c.466dupC, both of which are predicted to cause frameshifts leading to loss of repressor function and overexpression of the MmpL5 efflux pump (Sonnenkalb et al., n.d.; Snobre et al., 2024). Delamanid resistance–associated mutations were detected in *ddn* (c.223delG and p. Glu118*, both likely resulting in truncated, nonfunctional proteins) and *fbiC* (c.2565_*56del…), which is involved in cofactor F_420_ biosynthesis (Liu et al., 2022). These genetic alterations highlight multiple pathways to resistance, some of which may arise under selective pressure from prior drug exposure or cross-resistance mechanisms. Together, these findings indicate an emerging threat of advanced drug resistance in the region, underscoring the urgent need for ongoing genomic surveillance and the implementation of individualized treatment regimens informed by whole-genome sequencing or comprehensive drug susceptibility testing.

The clustering rate of 29.41% observed in this study is consistent with reports from other Central Asian countries, where active transmission is the primary driver of MDR-TB (Auganova et al., 2023; Engström et al., 2019). In contrast, Western European countries tend to report lower clustering rates, suggesting reactivation plays a larger role there (Guthmann and Haas, 2019; Walls and Shingadia, 2007). In Kazakhstan, especially in densely populated areas like Almaty, the high clustering rate and identification of multiple geographically and temporally linked clusters support the hypothesis of sustained community transmission.

Cluster C18, the largest identified, included strains spanning both years and predominantly affected patients residing within a 5 km radius. Intra-cluster evolution was observed, such as the progressive acquisition of resistance mutations in clusters C7 and C20. This indicates that some strains are not only being transmitted but are also evolving resistance *in situ*—possibly due to incomplete or ineffective treatment.

Spatial analysis revealed that 59.1% of clusters included patients from different regions, highlighting the potential role of inter-regional migration or mobility in the dissemination of *M. tuberculosis* strains. In some clusters, patients shared identical or nearly identical addresses, emphasizing the need to strengthen TB control in high-density housing or communal living environments.

To further explore the relationship between geographic proximity and genetic similarity of *M. tuberculosis* strains, we conducted a Mantel test using pairwise SNP distance matrices and geographic coordinates of patient residences. Initial analysis did not reveal a statistically significant correlation (Mantel r = 0.06173, *p* = 0.1628). However, a more detailed spatial autocorrelation analysis using the Mantel correlogram uncovered significant correlations at specific distance classes. Significant positive correlation was observed in the first distance class (D.cl.1; Mantel r = 0.0634, *p* = 0.041), indicating that patients living very close to each other (within ~5 km) were more likely to carry genetically similar strains. Suggesting spatial structuring of genetic diversity that may reflect complex patterns of TB transmission or mobility within and between communities. These findings indicate that while a broad correlation between geographic and genetic distance was not evident, spatial structure at finer scales does exist, supporting the hypothesis of localized transmission within the Almaty area.

The predominance of transmission-driven MDR-TB, the high proportion of isoniazid-monoresistant strains, and the emergence of pre-XDR/XDR-TB point to substantial gaps in current TB control measures in Kazakhstan. Strengthening contact tracing, enhancing community-based case finding, and introducing WGS-based surveillance in routine diagnostic workflows are crucial next steps (Burke et al., 2021; MacPherson et al., 2024). Special attention should be given to newly diagnosed cases and their close contacts, as the majority of clustered strains were found among new cases, indicating undetected chains of transmission.

Furthermore, molecular tools that can detect INH resistance should be prioritized to capture the full resistance spectrum. Incorporating WGS into national TB programs can enhance outbreak detection, guide individualized therapy, and provide real-time epidemiological insights.

Several limitations must be acknowledged. First, our analysis was restricted to culture-positive cases, potentially underestimating the burden of transmission from culture-negative patients. Second, we lacked detailed sociodemographic data (e.g., incarceration, homelessness, drug use) that may contribute to TB transmission risk. Lastly, as isolates were collected from a single national reference center, regional disparities in strain diversity and resistance patterns may have been missed.

This study provides a detailed genomic snapshot of the *M. tuberculosis* epidemic in Kazakhstan, revealing high levels of drug resistance, ongoing community transmission, and the dominance of a highly resistant lineage 2.2.1 strain. These findings highlight the urgent need for comprehensive TB control strategies incorporating WGS, rapid molecular diagnostics, and targeted interventions. Detecting isoniazid resistance—particularly given its high monoresistance rate (14%)—should be prioritized in clinical practice. As XDR-TB strains begin to emerge, national preparedness must include enhanced diagnostics, optimized treatment regimens, and regional collaboration to prevent the further spread of drug-resistant TB.

## Data Availability

The datasets presented in this study can be found in online repositories. The names of the repository/repositories and accession number(s) can be found here: https://www.ncbi.nlm.nih.gov/, PRJNA1277026.

## Footnotes

^1^ https://itol.embl.de/
